# Banana aphid, *Pentalonia nigronervosa* (Hemiptera: Aphididae): ecology and implications for the integrated management of banana bunchy top disease

**DOI:** 10.3389/finsc.2026.1883891

**Published:** 2026-08-05

**Authors:** Geofrey Ogwal, Walter Ocimati, Elizabeth Kearsley, Guy Blomme

**Affiliations:** 1 Bioversity International, Kampala, Uganda; 2 BlueGreen Labs, Melsele, Belgium; 3 Bioversity International, Addis Ababa, Ethiopia

**Keywords:** aphid dispersal, banana bunchy top virus, de-trashing, integrated pest management, soapy spray, winged aphids

## Abstract

**Introduction:**

Banana bunchy top disease (BBTD), caused by the banana bunchy top virus (BBTV), poses a growing threat to banana production in Africa and is spread locally by the banana aphid, *Pentalonia nigronervosa*. Effective BBTD management requires better understanding of aphid habitat, dispersal, response to farm operations, and practical aphid vector and disease control options.

**Methods:**

This study integrated field assessments, field experiments, and a laboratory trial in Uganda to characterize aphid ecology and refine integrated pest and disease management.

**Results:**

Aphids were highly cryptic, occurring mainly between leaf sheaths and on lower and middle pseudostem sections. Though winged aphids were mostly associated with larger aphid colonies they occurred across all colony sizes, indicating that even small colonies may contribute to BBTV spread. Sticky traps showed winged aphids to actively disperse throughout fields, with flights often extending beyond adjacent mats. Mechanical disturbance, simulating farm activities such as de-trashing and roguing, strongly increased movement of both winged and wingless aphids and occasionally flight, which could potentially increase BBTV transmission risk in case aphids are viruliferous. Aphids persisted on cut and disposed pseudostem pieces, especially under shaded conditions, but not on corms. In laboratory assays, aphids preferred leaf tissue, although field colonies were mainly pseudostem-associated. Spraying with a 1% laundry soap solution rapidly reduced both winged and wingless aphid populations within 30 minutes to two hours, with populations remaining low for up to two weeks on de-trashed plants.

**Discussion:**

These findings show that BBTD management should target hidden pseudostem colonies, minimize aphid dispersal during plant disturbance, apply soap spray before or immediately after de-trashing infected plants or infected mat removal, and rapidly bury infected pseudostems and leaves while drying corm pieces separately. The study provides practical refinements for smallholder-oriented BBTD integrated management.

## Introduction

Banana bunchy top disease (BBTD) constitutes a major constraint to banana production worldwide, causing significant yield losses that threaten food security (1). The causal agent, banana bunchy top virus (BBTV; species *Babuvirus musae*, family *Nanoviridae*; 2), is ranked among the top 100 invasive species globally due to its high transmissibility and devastating effects on banana [*Musa* spp.] (3, 4). The high transmission potential of BBTV stems from a coupled transmission involving human-induced movement and effective natural vector activity. Specifically, movement of infected planting material introduces BBTV to new fields and regions, while the banana aphid *Pentalonia nigronervosa* Coquerel (Hemiptera: Aphididae), prevalent in most banana producing regions, efficiently acquires and spreads BBTV locally within fields and between neighbouring farms (5, 6). The efficient spread of BBTV by banana aphids, compounded by a limited number of practical and highly effective control options for managing aphid populations, especially in smallholder banana production systems, renders BBTD management challenging.

The banana aphid, like many other aphid species, is cryptic, often concealed within host plant leaf sheaths (7, 8). They can occur as few individuals to large colonies of thousands, composed of a mix of unwinged and winged individuals (9). Winged individuals are critical in the spread of BBTV, as they move more efficiently from plant to plant. While the majority of aphid colonies are unwinged, production of winged morphs is generally induced by tactile stimulation of unwinged asexual aphids (10). As such, the size of the aphid colony may influence the formation of winged morphs, as dense populations create crowding increasing tactile stimulation that triggers alate production (10, 11). Other environmental cues for the production of winged aphids include host plant quality, interspecific interactions (such as with predators) and other abiotic stresses (9, 11–13). Nevertheless, triggers for winged banana aphid development and the habitat and behaviour of winged banana aphids remain poorly understood, particularly in the context of aphid management.

Banana aphid management within a BBTD Integrated Pest and Disease Management (IPDM) framework centers on preventing the dispersal of winged aphids to curb virus transmission (14). When BBTD is detected, targeted interventions such as insecticide sprays or systemic injections can be applied to infected mats before removal to prevent aphid movement to healthy plants (14). Despite their efficacy, the use of insecticides is often restrictive for smallholders through limited availability and high cost, in addition to environmental and health concerns (15). The quick burial of infected plant debris is an alternative means of removing viruliferous aphids from the field. Over a short timeframe, leaves and pseudostems need to be chopped up, positioned in a shallow pit, which is then covered by a thin layer of soil. This approach serves as a vital, low-cost method to trap and eventually kill all winged aphids and thus halt further pathogen spread (14, 15). Other cultural practices, such as removing old and dried out leaves, may also help in suppressing aphid population growth but are not general BBTD management recommendations (16). Overall, current BBTD management protocols offer minimal practical advice for controlling aphid vectors, leaving smallholders with few established options for effective BBTD management. This could be attributed to important knowledge gaps on the ecology of the banana aphid.

This study utilized field assessments, field experiments, and laboratory trials to characterize banana aphid populations in their natural environment and evaluate the impact of aphid management practices on aphid populations. With a primary focus on winged aphids, the research addressed six key objectives: (i) characterizing aphid population dynamics and their distribution across the banana mat; (ii) assessing the movement of winged aphids within the field; (iii) evaluating the impact of plant disturbance on aphid activity; (iv) examining the formation of winged aphids on decaying plant material; (v) aphid preferences for different banana plant parts; and (vi) assessing the effectiveness of soap spray as a sustainable alternative to chemical insecticides. The findings of this study inform practical refinements to BBTD IPDM.

## Materials and methods

All field assessments, field experiments, and laboratory trials were conducted at the National Agricultural Research Laboratories (NARL; 0°24′8.51” N, 32°31′39.18” E) in Kawanda, Uganda.

### Mapping aphid colonies across banana plant parts and growth stages

The first aphid assessment was conducted between June 19 and July 22, 2025, in three banana fields, each measuring approximately 2, 000 m² (0.5 acres), and containing at least 150 banana mats. Banana mats containing 1–4 plants were spaced at approximately 3 m × 3 m. A banana mat refers to a cluster of plants physically interconnected at the rhizome level. The three fields each serving as a replicate consisted of East African highland banana (*Musa* AAA-EAHB genome) cultivars. Each field was divided into six subplots that served as sampling strata, with each subplot having at least 25 banana mats. The 25 mats per subplot provided an adequate spatial coverage and a sufficient pool from which to select aphid-infested plants. Ten aphid infested banana plants, each from a different banana mat, were purposively selected per subplot for aphid enumeration. The developmental stage of each of the 10 sampled plants was recorded and categorized as peeper, water sucker, sword sucker, maiden sucker, or mature plant (**Supplementary Figure 1**). A total of 180 plants (60 plants per field) were thus sampled.

On each selected aphid-infested banana plant, aphid colonies were inspected *in situ*, and wingless and winged aphids in both open spaces and cryptic locations were enumerated separately. An aphid colony was defined as a distinct aggregation of aphids occurring on a specific plant part or location. For all colonies, aphids were counted in a systematic order to minimize omission or double counting. Open spaces were considered as those inside the funnel or cigar leaf, the point at which the petioles of the youngest leaves intersect, on the fresh pseudostem, and on pseudostem sections hidden under dried leaves (i.e., old leaves that hang like a ‘skirt’ around the pseudostem) (**Supplementary Figure 1**). Cryptic locations were considered as those in between old and fresh leaf sheaths and points where parts of the un-opened (un-furled) cigar leaf overlap. The old banana leaf sheaths were carefully opened to expose aphid colonies with minimal disturbance. De-trashing was not carried out in this trial. De-trashing refers to the removal of all leaf sheath sections that are dried up covering fresh pseudostems and dried out or partially dried leaves that hang like ‘a skirt’ around the plants’ stem. Aphid presence was also assessed on dried leaves. The time of day of aphid enumeration, i.e. morning, afternoon or evening, was recorded.

The distribution of winged and wingless aphid colonies along the pseudostem was also assessed on each plant. The presence of aphid colonies was recorded on the i) lower/bottom 30% pseudostem, ii) middle 30% pseudostem, and iii) upper 30% pseudostem sections, and iv) leaf level (i.e., the point at which the petioles of the youngest leaves intersect).

### Assessing aphids on the lower pseudostem section near the soil line to inform ‘roguing practices’

Banana ‘roguing’ in the context of BBTD management refers to the complete uprooting and destruction of infected or suspected infected banana mats/plants to reduce sources of BBTV inoculum and limit further aphid-mediated spread. To inform the practice of banana roguing, aphid (winged and wingless) presence and abundance at the lower/bottom 10 cm pseudostem section of maiden suckers starting from soil level was further assessed on 100 East African highland banana plants across two separate banana fields on August 5-6, 2025. For this study, each field was divided into five subplots with at least 20 mats, subplots serving as sampling stratum nested within the fields. Ten plants with multiple aphid colonies, each from an independent mat, were randomly sampled per subplot. The individual banana plants served as the sampling units, while the fields represented the highest spatial clustering level. For each plant, the presence, number, and location of winged and wingless aphids in either cryptic locations or open spaces was documented. The cryptic areas, such as the ones covered by dry leaf sheaths, were carefully and slowly opened for aphid enumeration to minimize disturbance of colonies.

### Tracking field-level movement of winged aphids using sticky traps

To monitor the within-field movement of winged banana aphids, yellow sticky traps were deployed between banana mats. Traps were installed in two East African highland banana fields, each containing at least 120 banana mats and a high number of winged aphids. Each field was divided into 5 subplots of 12–15 m^2^ that served as spatial sampling strata, with 4 traps installed in each subplot as repeated measures nested within the fields. The traps (25 x 10 cm, Biobet, Sustainable Crop Management) were labelled and pinned with thumbtacks to 60 cm cut wooden sticks [pole or peg], at a height of 45 cm above ground, and at 30 cm distance from a banana mat pseudostem base. The sticks were spaced at a minimum distance of 10 m.

On the day of setting the yellow sticky traps (July 24, 2025), day 1, each trap was installed at 7.00 a.m. and subsequently monitored at 10.00 a.m., 2:00 p.m., and 6:00 p.m. Subsequently, the traps were monitored at the same time intervals, at 2-day intervals, over a one-week period (i.e., at 4 different times). Data were collected on the cumulative total number of trapped winged aphids on the yellow sticky traps at each of the various times of the day for the sampling days. Data were also collected on number of winged and wingless aphids on the plant closest to the yellow trap at the start of the trial and at intervals corresponding to the sticky trap monitoring schedule.

### Testing whether plant disturbance triggers aphid movement

This field experiment assessed the response of banana aphids (winged and wingless) to disturbance through manual shaking of banana plants. East African highland banana plants were manually disturbed to mimic chopping of pseudostems and/or manual carrying of BBTD-infected plants/or plant pieces, a core practice for BBTD management, or to mimic plant movements (swaying, bending, and/or shaking) that may occur during strong wind or storm events. Disturbance was created by shaking the plant non-stop for 15 seconds. The plants were disturbed at separate times of the day, i.e. in the morning (low but increasing heat and solar radiation), in the afternoon (high temperature and irradiation [peak solar radiation]) and in the evening (moderate but decreasing temperature and radiation).

The experiment was conducted in two fields, each divided into 2 subplots (i.e. spatial sampling strata) each containing 30 mats. The individual banana plant was the experimental unit because the disturbance treatment was applied independently at the plant level, while the subplots and fields represented nested spatial grouping levels considered during model evaluation. Within each subplot, five different plants were disturbed while another five were left undisturbed as controls during three daily time windows: morning (8:00–11:00), afternoon (12:30–15:00), and evening (16:00–18:30). Different plants were selected for disturbance during each time window. As such, a total of 120 plants (60 plants per field) with aphid colonies containing at least one winged aphid, were purposively sampled. Selected plants had heights varying between 40 and 250 cm. All winged and wingless aphids in the colonies were recorded before and after the applied disturbance. The movement of aphids after shaking was then observed visually on the plant surface, categorized as (0) not moving or (1) moving, and recorded separately for winged and wingless aphids. The furthest distance (cm) moved by an aphid from the original aphid colony position on the plant surface was measured. The recorded distance represented the maximum observed movement for each aphid type, rather than the mean distance moved by each individual aphid. In the controls [i.e., unshaken] the general movement of the aphids was recorded. The activity of aphid tending ants was also observed following the disturbance.

### Aphid survival on discarded banana plant material

In this field experiment, a total of 20 East African highland banana pseudostem sections, each measuring 50 cm in length, and a total of 20 corm sections, each measuring approximately 5 × 12 cm^2^ were cut off from plants with multiple aphid colonies, on August 6, 2025. Ten of each plant part type section were randomly selected and placed under shaded conditions (i.e., under large trees), while another 10 were placed in areas exposed to sunlight in the field. For each of the two treatments, the cut pseudostem and corm sections were each placed into four batches (considered replicate), with each batch containing two or three pieces. Each individual batch was treated as the experimental unit, while the individual pseudostem or corm sections within a batch were treated as subsamples. The pseudostem sections had large aphid colonies while no aphids were observed on the corm tissues at harvest. The pseudostem and corm sections were monitored for 3 weeks, at 2 to 3–day intervals. Data on size of aphid colony (number of wingless aphids), and the presence and number of winged aphids at each time interval were collected from each pseudostem or corm section per batch.

### Aphid preference for and performance on different banana tissues *in vitro*

Aphid preference for different parts of banana plants, i.e., leaf, pseudostem, and corm, was determined through ‘no-choice’ and ‘choice’ laboratory experiments conducted in transparent plastic jars (9 cm base diameter, 14 cm height, 800 mL capacity). Jar lids were perforated by making 8–10 small holes of 1 mm diameter to provide ventilation. The lids and the base of the jar were lined with tissue paper to respectively, prevent aphid escape and absorb excess moisture. Leaf and pseudostem tissue was collected from mature disease-free East African highland banana plants at NARL. Leaf tissue (containing the midrib) was collected from leaves > 1m long, while pseudostem tissues were obtained from inner fresh leaf sheaths. Corm tissue was collected from maiden suckers. Tissue sections of approximately 5×12 cm were placed standing in the jars, either singly (no-choice experiment), or paired next to each other (choice experiment) in combinations of leaf with pseudostem, leaf with corm and pseudostem with corm. Each treatment (tissue) was replicated in six separate individual jars, with each jar serving as an independent experimental unit.

An initial population of 20 adult aphids, collected from the field, was introduced into each jar using a fine moistened nylon brush (No. 1). The aphids were distributed evenly across the different sides of a plant tissue. The jars with aphids were maintained in the laboratory at a temperature of 26 ± 2°C, 67 ± 7% relative humidity and 12 h photoperiod. Aphids were monitored for a period of 15 days. The number of aphids (combining nymphs and adults) in each jar (distinguishing the tissue samples), both alive and dead, were counted at 3-day intervals. To compare population growth rates between each time step, a 3-day growth rate was derived, assuming a rate of change between each time step of *x2*(*t*) = *x*_1_ × (1 + *r*)^*t*, with *x2*(*t*) as the population after time t, *x*1 the initial population, r the growth rate, and t the 3-day time interval. Within each treatment, a similar growth rate between varying time steps would indicate a balanced population growing at a steady rate. A positive growth rate (r > 0) indicates population-level reproduction is larger than population-level mortality, while a negative growth rate (r < 0) indicates population-level mortality exceeds population-level reproduction. The 3-day mortality at each time step was derived as the newly deceased aphids divided by the total living aphid population at the previous 3-day time interval.

### Testing soap spray as a low-cost aphid control option

This experiment was conducted in five different banana fields, each containing at least 150 banana plants with many winged aphids. The fields consisted of East African highland banana cultivars, consistent with the other field experiments in this study. Each field functioned as an independent replicate, while the individual banana plant was the treatment unit to which the soap or control treatment was applied. Four maiden suckers and/or mature plants per field, each with aphid colonies containing at least 2–3 winged individuals, were purposively sampled and tagged with a labelling ribbon. On each tagged plant, dried out leaves, fresh pseudostem parts, unfurled leaves, green leaf petioles, and the space between leaf sheaths were examined and the presence and number of winged aphids recorded and thereafter de-trashed. De-trashing aimed at allowing the spray to cover all the pseudostem surface and to reach all the aphids. After de-trashing, the number of winged and wingless aphids was re-recorded. Next, two plants per field were sprayed using a 1% laundry bar soap (brand: White Star) solution [made by dissolving 10 g soap in 990 mL distilled water ≈ 80 °C], while the other two were kept as unsprayed controls. Spraying was done using a 20 L knapsack sprayer (MIRÒ, BI. VI. IRRORAZIONE SNC, Italy), having an adjustable nozzle. All plants were monitored at 30 minutes after spray, and subsequently at 2–hour intervals over an 8–hour period. Additionally, all plants were monitored at 7-day intervals over a 6-week period to assess aphid re-infestation or population recovery. No additional soap sprays were applied over the 6-week monitoring period. The weekly observations therefore reflect aphid population changes following a single initial soap spray application. At each post-treatment assessment interval, data were collected on the number of living winged and wingless aphids, as described above.

### Data analysis

The unit of analysis differed with the study design and the level at which observations or treatments were applied as described in each study sub-section above. Mixed-effects binomial logistic models and negative binomial generalized linear mixed models (GLMMs) or generalized linear models (GLMs) were employed to predict aphid presence and abundance throughout the study experiments. The statistical framework was adapted to accommodate heterogeneous experimental designs: some studies involved repeated observations on the same plants, traps, or pseudo-stem sections over time, while others consisted of single-timepoint measurements with spatial replication only.

For experiments involving longitudinal sampling, temporal dependency was addressed through two complementary strategies. First, days since initiation was included as a continuous fixed-effect covariate where applicable, allowing the model to partition variation attributable to temporal progression independently from treatment effects. Second, the observation time window was optionally treated as a categorical factor to avoid assumptions of linear temporal trends. Where supported by model comparison, random intercepts were specified for the relevant repeated-measure units, such as a individual plants, traps, and batches of cut pseudostem and corms, nested at spatial grouping levels such as subplots and fields. This accounted for non-independence among repeated observations from the same unit and among observations clustered within the same spatial grouping level. Experiments without repeated measures omitted these temporal covariates.

Across the field-based study components, candidate random-effects structures reflected the spatial design. Subplots were treated as sampling strata nested within fields, while fields represented the highest spatial clustering level. Random effects were retained only when they improved model fit, as determined using likelihood-ratio tests and AIC comparisons. This hierarchical random approach accounted for the clustering of observations within subplots and fields without treating within-field observations as independent field-level replicates.

The inclusion of random effects (subplots nested in fields, or separately) was justified through likelihood ratio tests comparing the mixed-effects baseline to a fixed-effects intercept-only model (with boundary correction applied for variance component testing; p <.05; 17) and by demonstrating a lower Akaike Information Criterion (AIC; 18). Residual autocorrelation was assessed visually using ACF plots where sample sizes permitted. No substantial temporal autocorrelation beyond what was captured by the random structure remained in final models.

For count-based response variables (e.g., wingless and winged aphid abundance, colony size), negative binomial GLMMs or GLMs with a log-link function were fitted instead of linear mixed models to appropriately handle the distributional properties of count data. Poisson models were initially considered but consistently failed to provide better fits than negative binomial alternatives, as confirmed by likelihood ratio tests. Where random effects were not warranted (based on AIC and likelihood ratio tests), negative binomial GLMs were fitted without random intercepts. Model diagnostics for count models were performed using simulated residual checks via the DHARMa package (19), including quantile-quantile plots of scaled residuals, tests for overdispersion, and tests for zero-inflation. Zero-inflation components were retained only where statistically supported (p <.05); otherwise, simpler negative binomial specifications were preferred. For models without random effects, McFadden’s pseudo-R² was reported as a measure of model fit.

For the experiment measuring aphid movement in response to plant disturbance, with maximum distance as the continuous response variable, linear regression was fitted after confirming normality of residuals and homoscedasticity. Random effects were tested but not retained.

Binomial mixed-effects models were fitted using the glmer function from the lme4 package version 1.1.37 (20), while negative binomial GLMMs and GLMs were fitted using the glmmTMB function from the glmmTMB package version 1.1.10 (21). Linear mixed-effects models for non-count continuous response variables were fitted with the lme function from the nlme package version 3.1.168 (22, 23). Exhaustive model search evaluated all biologically plausible main effects (including aphid location, plant developmental stage, colony size, time of day, disturbance, treatment, days since initiation, and tissue type) with best-fitting models selected based on AIC. For the binomial models, effect sizes are reported as odds ratios, where a value greater than 1 indicates an increased probability of aphid presence relative to the reference level, while a value less than 1 indicates a decreased probability. For negative binomial models, effect sizes are reported as incidence rate ratios (IRR), where values greater than 1 indicate higher expected counts relative to the reference level and values less than 1 indicate lower expected counts. Where applicable, analysis of variance (ANOVA) was conducted after verifying assumptions of normality and homoscedasticity using the Shapiro–Wilk and Bartlett’s tests, respectively. All analyses were performed in R statistical software version 4.5.2 (23).

## Results

### Mapping of aphid colonies across banana plant parts and growth stages

Aphids were observed on all plants, as small groups of individuals or as colonies. Ninety percent of plants contained both wingless and winged aphids, while 6% solely contained wingless aphids and 4% solely contained winged aphids. Colony size estimated using wingless aphid abundance, was dependent on the field (as random effect), the age of the plant and cryptic locations, while the time of day did not affect wingless aphid counts. Cryptic locations contained significantly more wingless aphids than open spaces (IRR = 3.02, p = <.001), and higher numbers of aphids were recorded on maiden suckers (IRR = 2.18, p = <.001) and sword suckers (IRR = 2.5, p < 0.05) (**Table 1**). Aphids were significantly more present on bottom and middle sections of the pseudostem (on 48% ± 23% of plants; 63% ± 22% of plants; respectively) compared to upper section of the pseudostem or on leaves (on 9% ± 11% of plants; 24% ± 17% of plants; F = 29.84, p <.001). No aphids were observed on dried out leaves.

**Table 1:** Best model fits for the prediction of the number of wingless and winged aphids.

(1) Number of wingless aphids			
Predictors	Incidence Rate Ratios	CI	p
(Intercept)	9.81	4.33 – 22.23	**<0.001**
Aphid location [cryptic]	3.02	1.67 – 5.47	**<0.001**
Development stage [maiden sucker]	2.18	1.15 – 4.15	**0.017**
Development stage [water sucker]	1.56	0.71 – 3.42	0.266
Development stage [sword sucker]	2.5	1.02 – 6.17	**0.046**
Development stage [peeper]	0.73	0.20 – 2.70	0.636
Random Effects*			
σ^2^	1.69		
τ_00_ Field	0.16		
ICC	0.09		
N Field	3		
Observations	360		
Marginal R^2^/Conditional R^2^	0.197/0.266		
(2) Number of winged aphids			
Predictors	Incidence Rate Ratios	CI	p
(Intercept)	0.41	0.29 – 0.58	**<0.001**
Aphid location [cryptic]	2.84	2.09 – 3.86	**<0.001**
Non winged aphid number	1.01	1.00 – 1.01	**<0.001**
Observations	360		
McFadden pseudo-R²	0.0812		

(1) Negative binomial generalized linear mixed model (GLMM) fit by maximum likelihood to predict wingless aphid counts on banana plants, with aphid location (open or cryptic locations) and plant development stage (mature, maiden sucker, water sucker, sword sucker, or peeper) as fixed effects predictors and field as a random intercept. (2) Negative binomial GLM with log-link to predict winged aphid counts on banana plants. Predictors include aphid location, wingless aphid number, and plant development stage. No random effect was retained for this model. Additional model diagnostics available in **Supplementary Figure 2** and **S3**.

*Random effects σ2, τ00 Field, ICC and N denote Residual Variance, Random Intercept Variance, intraclass correlation coefficient for mixed models and number of random effects groups, respectively.

Bold values in column labeled P indicate significant differences at p≤ 0.05.

**Figure 1:**
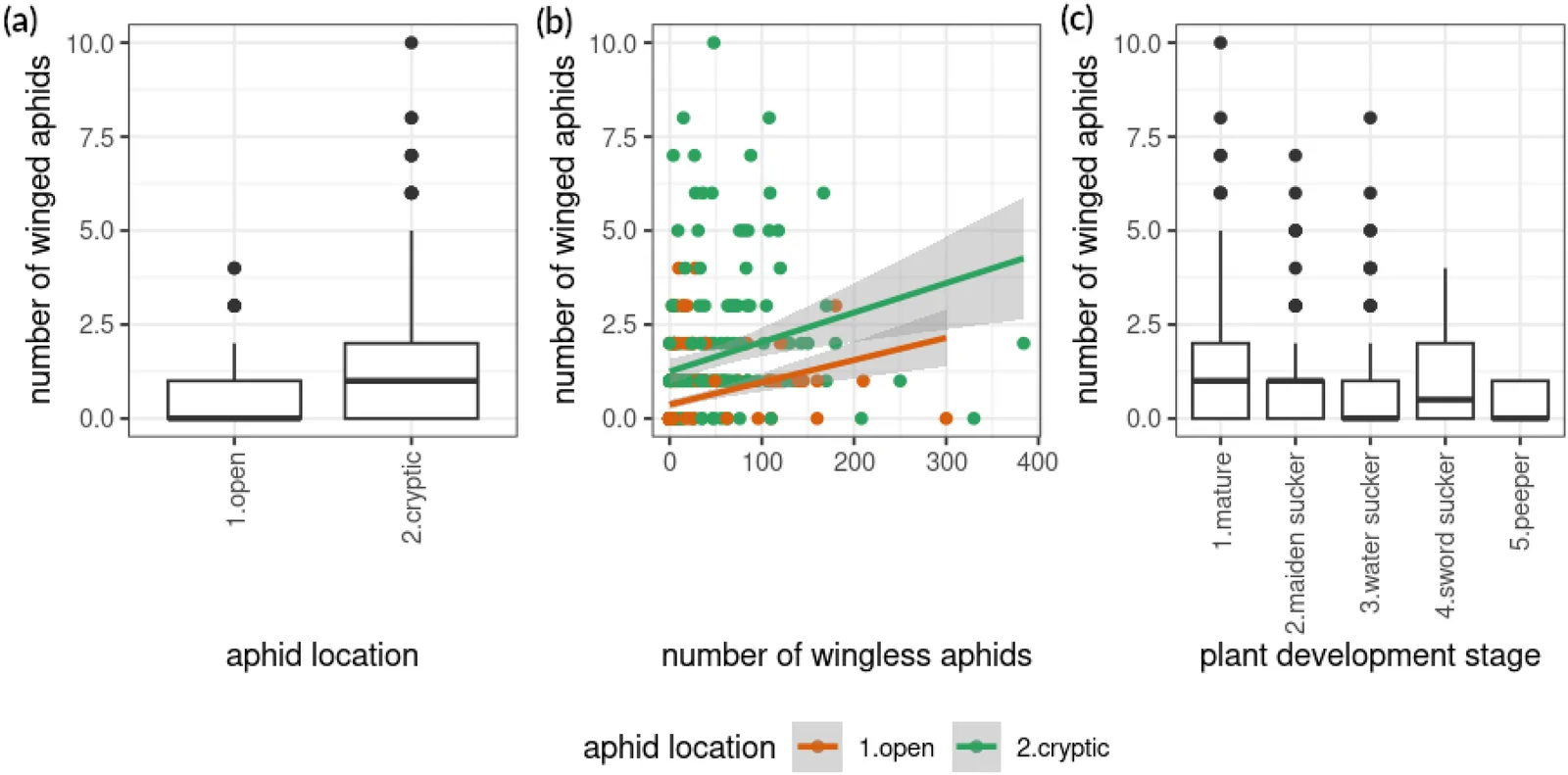
Number of winged aphids as dependent on **(a)** location, **(b)** wingless aphid colony size and **(c)** plant development stage.

Winged aphid presence was significantly related to wingless aphid presence (odds ratio 49.5, 95% CI [24.7, 111.2]), while no impact of time of day, plant development stage, or field assessed was observed. The number of winged aphids was significantly increased in cryptic locations compared to open spaces (IRR = 2.84, p = <.001) and increased with wingless aphid colony size (IRR = 1.01, p = <.001) (**Table 1**, **Figure 1**).

### Banana aphid assessment on the lower pseudostem near the soil line to inform roguing practices

Although all assessed plants contained aphids, 69% ± 15% had aphids on the bottom 10 cm of the pseudostem. On plants with aphids, the number of wingless aphids on the bottom 10 cm of the pseudostem were significantly increased in cryptic locations compared to open space (IRR = 2.28, p = <.05) (**Table 2**). No winged aphids were observed on the bottom 10 cm of the pseudostem.

**Table 2:** Best model fits for the prediction of wingless aphid counts on bottom 10 cm of the pseudostem of banana plants.

Number of wingless aphids on bottom 10 cm of pseudostem			
Predictors	Incidence Rate Ratios	CI	p
(Intercept)	9	4.94 – 16.39	**<0.001**
aphid location [cryptic]	2.28	1.17 – 4.45	**0.016**
Observations	69		
McFadden pseudo-R²	0.0091		

Negative binomial GLM with log-link, with aphid location (open or cryptic locations) as predictors. No random effect was retained for this model. Additional model diagnostics available in **Supplementary Figure 4**. Bold values in column labeled P indicate significant differences at p≤ 0.05.

### Tracking of the field-level movement of winged aphids using sticky traps

The number of winged aphids trapped on the yellow sticky traps did not vary with time of day of assessment (7:00-10:00, 10:00-14:00 or 14:00-18:00), the day of the week of sampling, or the number of winged or wingless aphids on the nearest banana plant (**Supplementary Table 1**; **Supplementary Figure 5**). Solely a subplot level difference was observed, with two subplots having significantly higher numbers of trapped winged aphids over the course of a day across 4 traps (between 7:00 and 18:00; 9.4 ± 4.3 winged aphids trapped per day; F = 6.5, p < 0.001) compared to other subplots (1.9 ± 1.5 winged aphids trapped per day). Overall, winged aphids trapped in a subplot per day varied between 0 and 17 aphids (median of 2).

### Effect of manual plant disturbance on banana aphid movement

On undisturbed plants, winged aphids were more likely to move than wingless aphids (odds ratio 6.12, 95% CI [1.34, 41.81]) and increased with the size of the aphid population (keeping other variables constant) (odds ratio 1.01, 95% CI [1.00, 1.03]) (**Table 3**). Aphid movement was marginally reduced in the afternoon and evening (**Table 3**). No dependency on fields or subplots as random effect was observed. The average furthest distance moved by aphids was 4.5 ± 4.2 cm, while no further effect of time of day, aphid colony size or aphid type was observed.

**Table 3:** Best model fits for the prediction of aphid movement.

(1) Movement on undisturbed plants			
*Predictors*	*Odds Ratios*	*CI*	*P*
(Intercept)	0.06	0.01 – 0.25	**0.001**
Time [2_Afternoon]	0.22	0.04 – 0.92	0.056
Time [3_Evening]	0.2	0.03 – 0.90	0.057
Number of aphids	1.01	1.00 – 1.03	**0.014**
Aphid type [winged]	6.12	1.34 – 41.81	**0.033**
Observations	120		
^#^R^2 Tjur^	0.172		
(2) Movement on disturbed plants and controls			
Predictors	Odds Ratios	CI	P
(Intercept)	0.07	0.02 – 0.16	**<0.001**
Disturbance [Disturbed]	15.29	7.52 – 33.62	**<0.001**
Time [2_Afternoon]	0.71	0.32 – 1.54	0.388
Time [3_Evening]	0.34	0.15 – 0.76	**0.01**
Number of aphids	1.01	1.01 – 1.02	**0.002**
Aphid type [winged]	3.1	1.47 – 6.81	**0.004**
Observations	240		
R^2^_Tjur_	0.331		
(3) Maximum distance moved on disturbed plants and controls			
Predictors	Estimates	CI	P
(Intercept)	-0.17	-1.79 – 1.44	0.834
Disturbance [Disturbed]	4.55	3.28 – 5.82	**<0.001**
Time [2_Afternoon]	-1.23	-2.79 – 0.32	0.12
Time [3_Evening]	-2.32	-3.88 – -0.77	**0.003**
Aphids count before shaking	0.02	0.00 – 0.03	**0.037**
Aphid type [winged]	2.81	1.38 – 4.24	**<0.001**
Observations	234		
R^2^	0.241		

(1) Logistic regression to model movement (0: not moving; 1: moving) of aphids on undisturbed plants, as predicted by the time of day (morning, afternoon, evening), aphid colony size, and aphid type (wingless and winged). (2) Logistic regression to model movement (0: not moving; 1: moving) of aphids on disturbed plants, as predicted by the disturbance (control and disturbance), the time of day, aphid colony size, and aphid type. (3) Linear regression to predict the maximum distance moved by an aphid, as predicted by disturbance, the time of day, aphid colony size, and aphid type. No random effects were retained for the development of these models.

^#^Tjur’s R^2^ (coefficient of discrimination) serves as a pseudo-R^2^ metric optimized for evaluating logistic regression models. Bold values in column labeled P indicate significant differences at p≤ 0.05.

**Figure 2:**
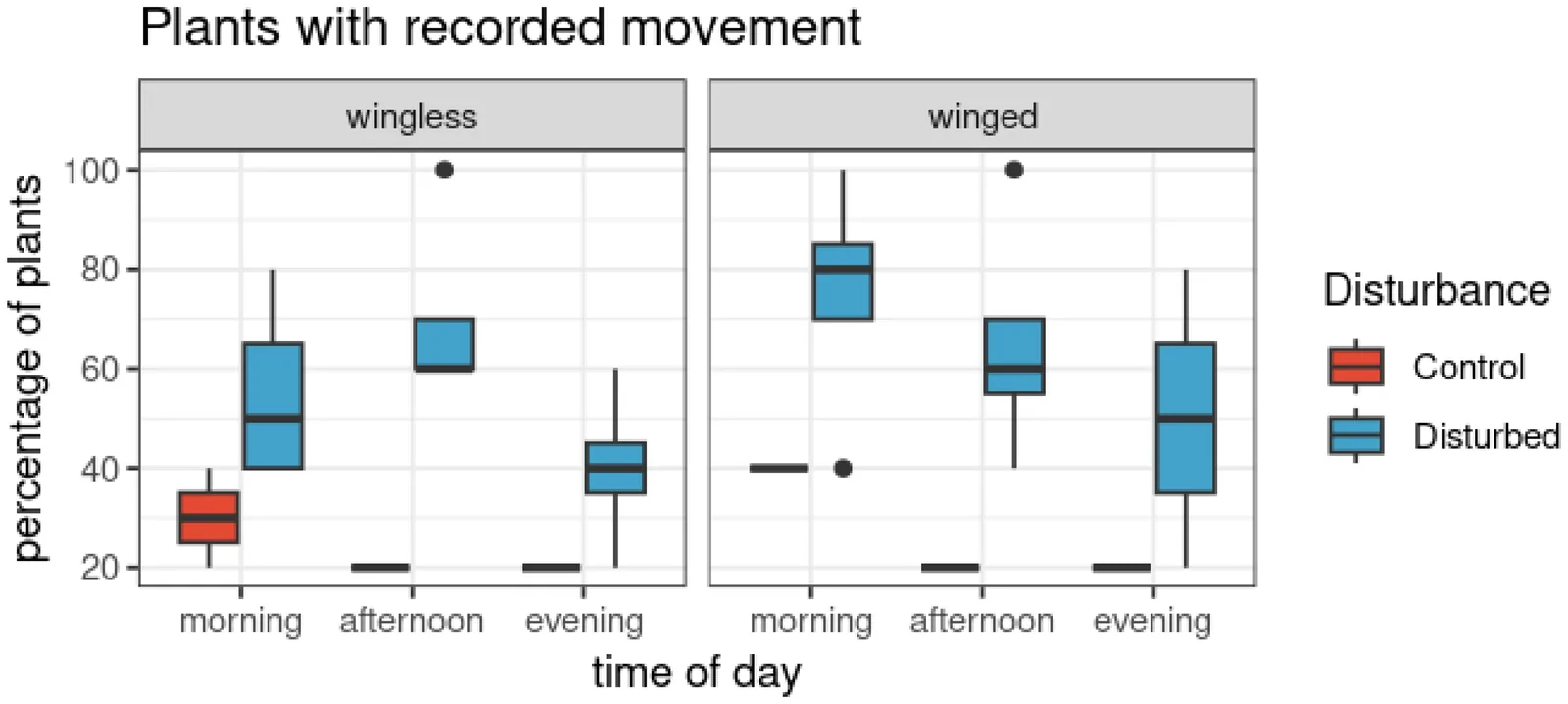
Percentage of plants (per subplot) with recorded movement of wingless and winged aphids on disturbed and undisturbed (control) plants during the morning, afternoon and evening.

Inflicting disturbance by shaking significantly increased aphid movement (odds ratio 15.23, 95% CI [7.52, 33.62]; **Table 3**, **Figure 2**). Movement was greater among winged aphids and increased with colony size but reduced during the evening (**Table 3**). The furthest distance moved was similarly increased by disturbance, increase in colony size and in winged aphids, while it reduced in the evening (**Table 3**). On disturbed plants, the average distance moved was 5.7 ± 5.5 cm by wingless aphids and 11.6 ± 8.4 cm by winged aphids; these estimates exclude flight distance. On 6 disturbed plants, winged aphids were observed to fly away, independent of the time of day.

Disturbance did not impact ant movement. Ant movement was marginally reduced during the afternoon (odds ratio 0.39, 95% CI [0.13, 1.09], p < 0.1).

### Aphid survival on discarded banana plant material

**Figure 3:**
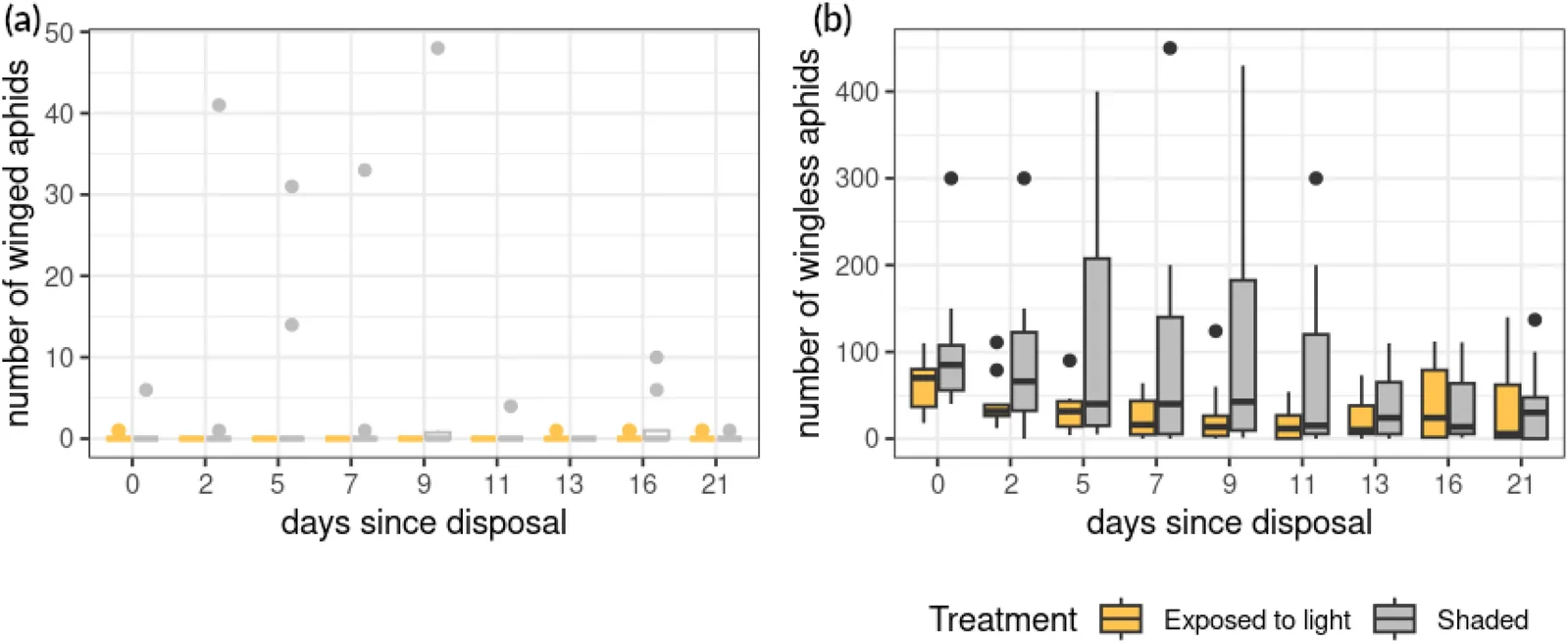
Number of winged **(a)** and **(b)** wingless aphids on disposed pseudostem sections in shaded or sunlight-exposed areas, over a 21-day period.

No banana aphids were observed on the banana corm sections at the onset of the trial and over the 21 days of the study, while aphids were observed on the pseudostem sections. Sporadic winged aphid occurrence was observed on the pseudostem sections, although no model predictors were retained for treatment (shaded vs exposed to sunlight) or days since plant section disposal (**Figure 3a**). One pseudostem section in a shaded area was observed to have a substantially increased number of winged aphids from day 2 to day 9, ranging from 31 to 48 winged aphids.

The number of wingless aphids depended significantly on treatment of the disposed pseudostem section, with sections in shaded areas showing increased numbers (IRR = 2.31, p = 0.001) (**Table 4**; **Figure 3b**). This treatment difference remained present until day 9. At day 11, marginal drops in shaded sections were recorded, and by day 13 significant reductions in wingless aphid counts were recorded on shaded sections.

**Table 4:** Negative binomial generalized linear mixed model (GLMM) fit by maximum likelihood to predict wingless aphids on disposed pseudostem section, as predicted by treatment (shaded vs exposed to sunlight), and days since disposal.

Number of wingless aphids			
Predictors	Incidence Rate Ratios	CI	p
(Intercept)	53.64	28.35 – 101.47	**<0.001**
Treatment [Shaded]	2.31	1.44 – 3.72	**0.001**
Days after initiation [2]	0.74	0.32 – 1.71	0.487
Days after initiation [5]	0.77	0.33 – 1.77	0.54
Days after initiation [7]	0.61	0.26 – 1.40	0.243
Days after initiation [9]	0.68	0.29 – 1.57	0.362
Days after initiation [11]	0.44	0.19 – 1.04	0.06
Days after initiation [13]	0.37	0.16 – 0.85	**0.019**
Days after initiation [16]	0.51	0.22 – 1.17	0.112
Days after initiation [21]	0.47	0.20 – 1.09	0.08
Random Effects			
σ^2^	1.03		
τ_00_ Batch	0.03		
ICC	0.03		
N Batch	8		
Observations	180		
Marginal R^2^/Conditional R^2^	0.201/0.223		

Additional model diagnostics available in **Supplementary Figure 6**. Bold values in column labeled P indicate significant differences at p≤ 0.05.

Field observations showed that decay of the pseudostem pieces was slow during the 21-day period due to persistent rainfall, with wilting occurring gradually, maintaining plant tissues in a condition favourable for aphid survival.

### Aphid preference for and performance on different banana tissues *in vitro*

**Figure 4:**
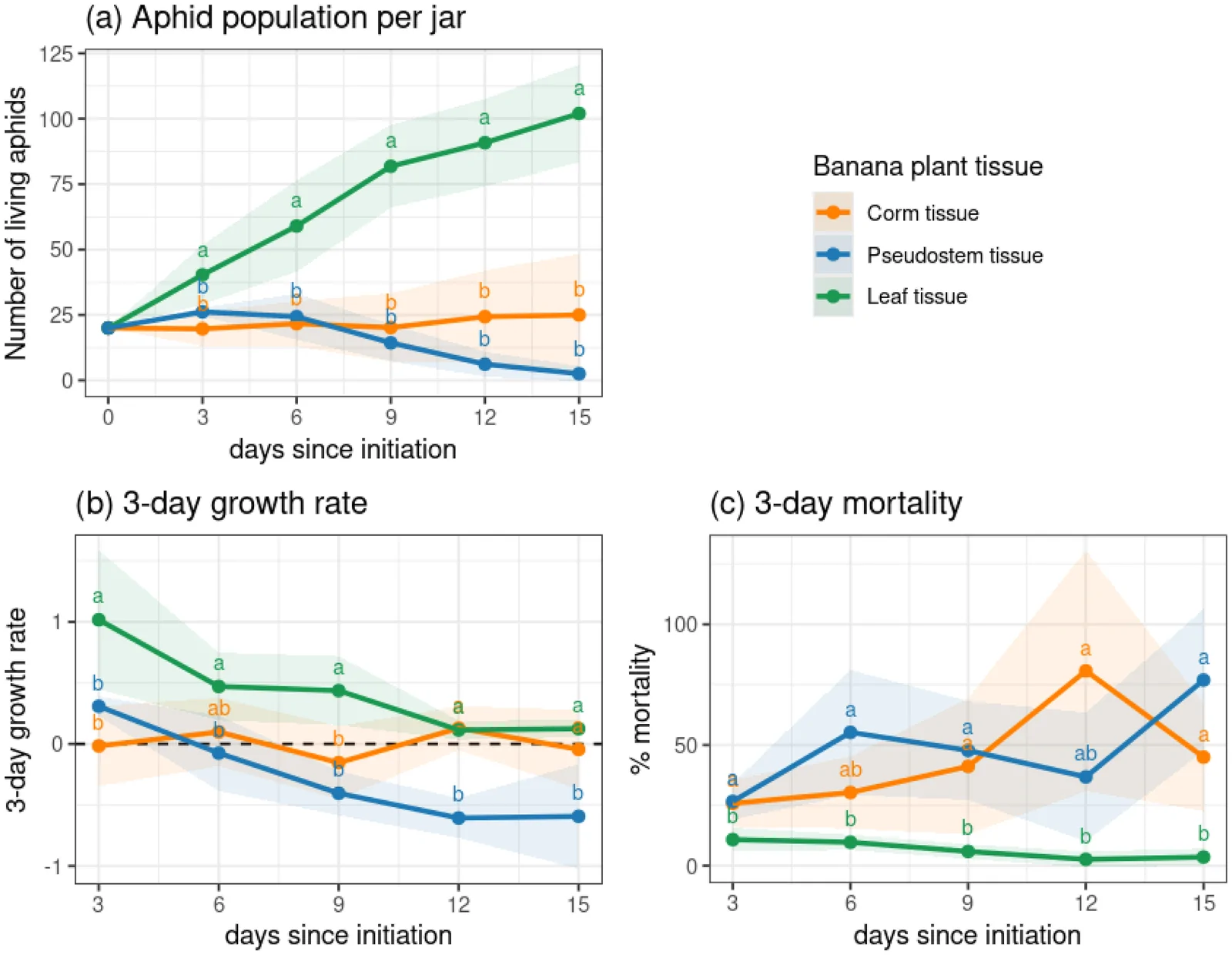
Number of living aphids per jar **(a)**, 3-day growth rate **(b)** and 3-day mortality rate **(c)**, over a 15 day period, and according to leaf, corm or pseudostem tissue, in the no-choice experiments.

Aphids showed a clear preference and population increase on leaf tissue. In the no-choice experiments, the population on leaf sections was significantly higher from day 3 onwards (F = 11.48, p <.001; **Figure 4a**). On leaf tissue, the 3-day growth rate remained higher for 9 days (F = 16.72, p <.001; **Figure 4b**), while mortality remained low during the 15-day monitoring period (F = 17.70, p <.001; **Figure 4c**).

**Figure 5:**
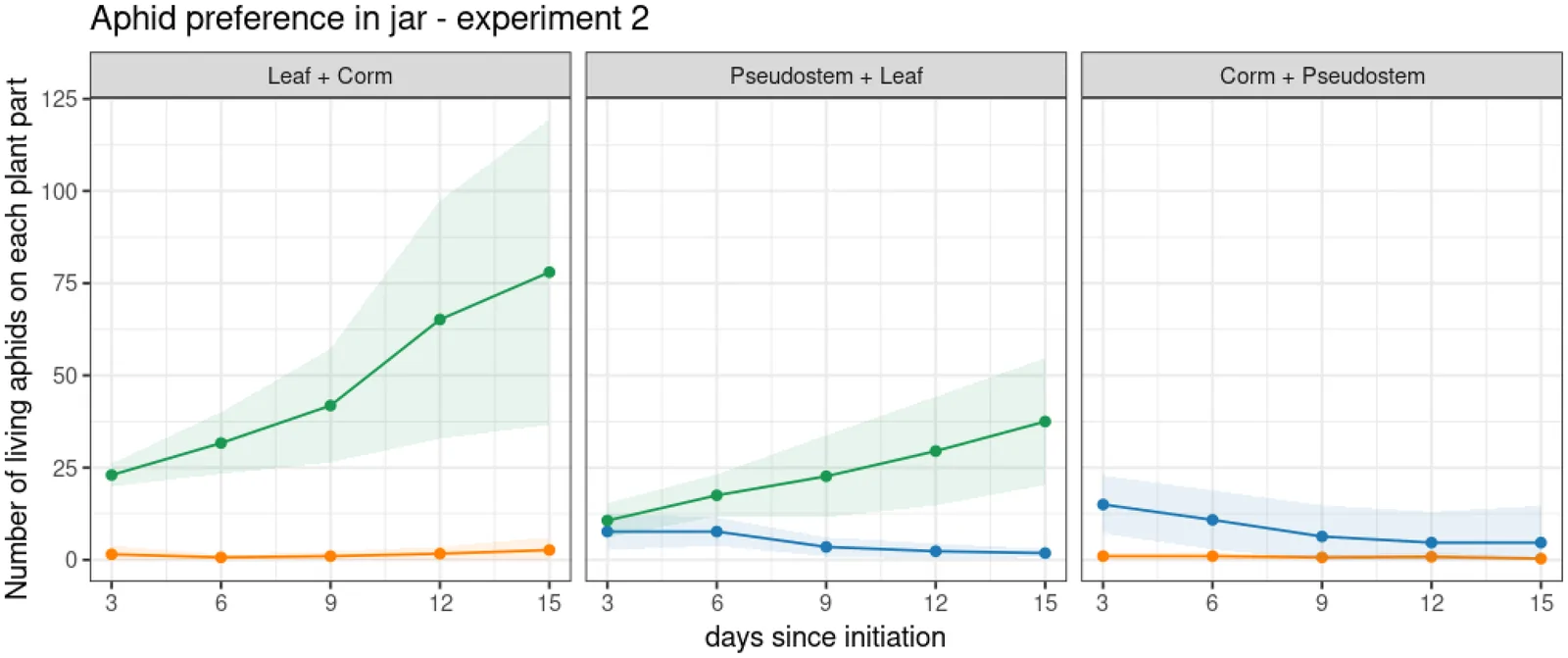
Number of living aphids per jar, by plant part, over a 15-day period, in the choice experiments.

Providing the aphids with a choice between banana plant tissue sections, a clear preference was observed for leaf tissue. When no leaf tissue was available, and the choice was restricted between pseudostem and corm tissues, a preference was observed for pseudostem tissue, although overall performance and population growth remained low (**Figure 5**).

### Testing soap spray as a low-cost aphid control

**Figure 6:**
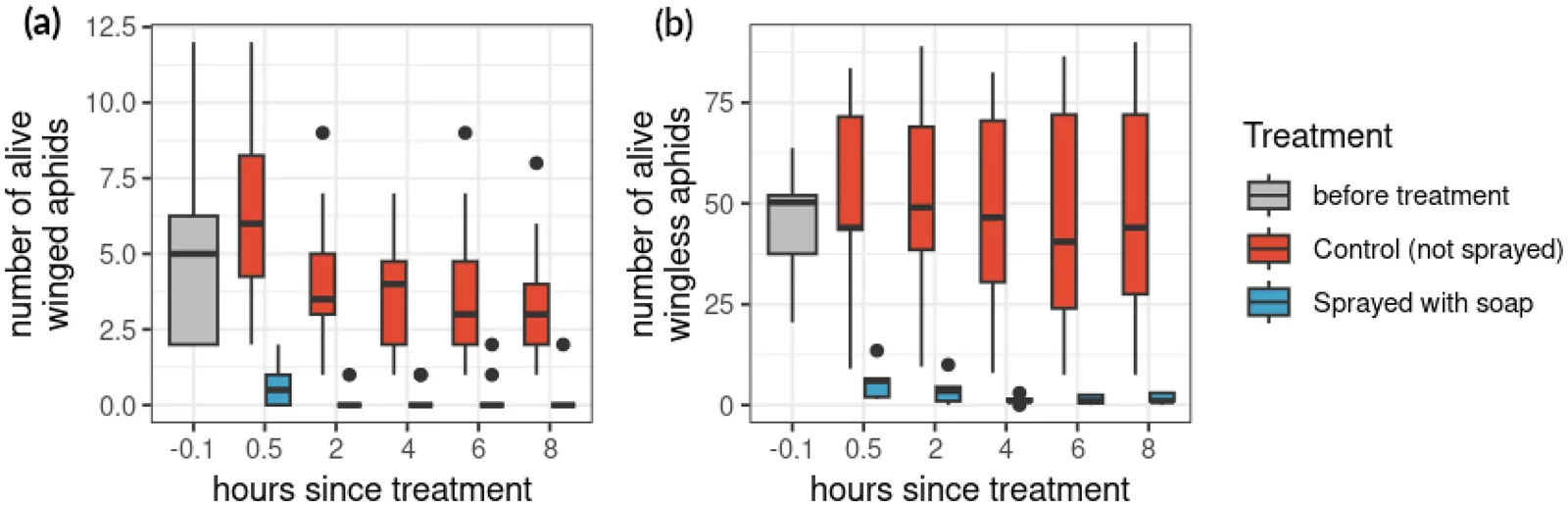
Evolution of **(a)** winged and **(b)** wingless aphid numbers on sprayed and non-sprayed plants. A timing value of -0.1 represents aphid counts taken immediately prior to treatment, whereas time 0 denotes the initiation of the treatment.

The number of observed winged aphids was significantly higher after de-trashing (F = 20.47, p < 0.001) due to exposure of previously hidden colonies, with an average 1.8 ± 1.2 winged aphids observed before de-trashing and 5.3 ± 3.2 after de-trashing. Because de-trashing exposed previously concealed aphids, post-de-trashing aphid counts were used as the treatment baseline to assess the impact of the soap spray treatment. The number of winged aphids reduced significantly due to the soap treatment (IRR = 0.07, p = <.001) (**Table 5**; **Figure 6**), with a significant reduction observed over the course of the monitoring hours (IRR = 0.93, p < 0.05). On treated plants, a clear drop in number of winged aphids was observed from 30 minutes to 2 hours post-application, while numbers remained low up to 8 hours post-application (**Figure 6**). An initial drop in number of winged aphids from 30 minutes to 2 hours in the de-trashed controls was also observed, suggesting that the exposure of aphids due to de-trashing, could have led to some winged aphids flying away within the 2 hours following de-trashing. Wingless aphids also reduced significantly over the first two hours post spray application (IRR = 0.07, p = <.001) (**Table 5**; **Figure 6**), with a further significant reduction during the remainder of the treatment day (IRR = 0.92, p < 0.01).

**Table 5:** Best model fits for the prediction of soap treatment on aphid numbers on the day of treatment.

(1) Number of surviving winged aphids			
Predictors	Incidence Rate Ratios	CI	p
(Intercept)	5.21	4.09 – 6.65	**<0.001**
Treatment [Control (not sprayed)]	1.06	0.73 – 1.54	0.748
Treatment [Sprayed with soap]	0.07	0.04 – 0.13	**<0.001**
Hours	0.93	0.88 – 0.99	**0.023**
Observations	120		
McFadden pseudo-R²	0.257		
(2) Number of surviving wingless aphids			
Predictors	Incidence Rate Ratios	CI	p
(Intercept)	43.85	21.68 – 88.69	**<0.001**
Treatment [Control (not sprayed)]	1.29	0.78 – 2.16	0.325
Treatment [Sprayed with soap]	0.07	0.04 – 0.11	**<0.001**
Hours	0.92	0.86 – 0.98	**0.008**
(Intercept)	5.42	3.31 – 10.88	
Zero-Inflated Model			
(Intercept)	0	0.00 – Inf	0.996
**Random Effects**			
σ^2^	0.03		
τ_00_ Field	0.48		
ICC	0.94		
N Field	5		
Observations	120		
Marginal R^2^/Conditional R^2^	0.813/0.989		

(1) Negative binomial GLM with log-link to predict winged aphid counts related to soap treatment. Predictors include treatment and hours since treatment. No random effect was retained for this model. (2) Zero-inflated negative binomial generalized linear mixed model (ZINB GLMM) fit by maximum likelihood to predict wingless aphid counts related to soap treatment, with treatment and hours since treatment as fixed effects predictors and field as a random intercept. A zero-inflation component was included to address excess zeros arising from complete aphid mortality following soap spray treatment. Additional model diagnostics available in **Supplementary Figures 7** and **S8**. Bold values in column labeled P indicate significant differences at p≤ 0.05.

**Figure 7:**
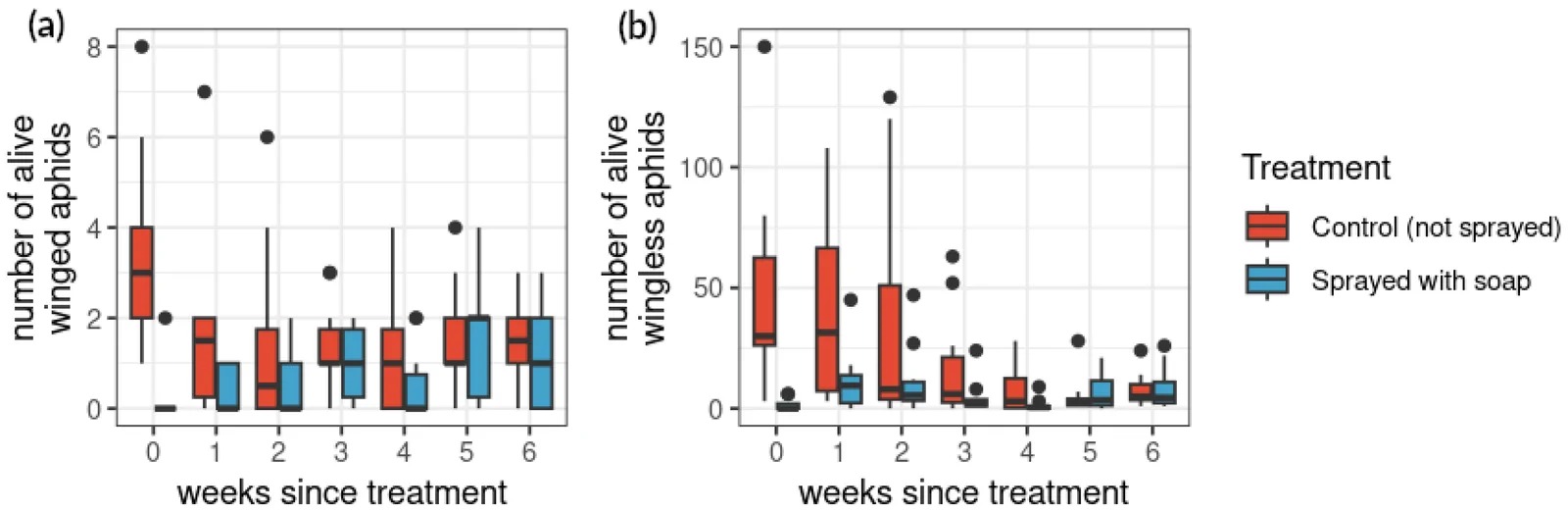
Evolution of **(a)** winged and **(b)** wingless aphid numbers during the 6-week period following the soap spray treatment.

Over the course of the 6-week observation period, the number of winged aphids remained low, indistinguishable from the control. Overall wingless aphid populations dropped marginally by week 3 and significantly by week 4 onwards in both control and treated plants (**Figure 7**).

## Discussion

Banana aphid population density was strongly associated with winged aphid abundance, supporting the hypothesis that crowding contributes to alate formation. This mechanism is well-documented in aphid biology, where tactile stimulation among wingless individuals trigger wing induction as a dispersal mechanism (10, 11, 24, 25). Nevertheless, our observation that winged aphids occur across the full range of colony sizes suggests that even small colonies can act as potential sources of winged vectors and transmission risk for BBTV in BBTD affected farms and landscapes. Consequently, even small aphid colonies should be considered during aphid surveillance and targeted management in BBTD affected mats, farms and landscapes.

The spatial distribution of banana aphids on banana plants demonstrates a distinct preference for cryptic locations, particularly between leaf sheaths in the lower and middle pseudostem sections. This finding corroborates established literature on aphid behaviour, in which concealment serves as a primary defence mechanism against predators and environmental stressors (26, 27). Notably, both winged and wingless individuals were aggregated in these protected spaces. These findings have important implications for scouting protocols, as aphid presence may be underestimated when inspecting only exposed plant surfaces.

Yellow sticky trap data further revealed substantial winged aphid activity throughout the field, with aphid captures occurring at all times of the day. The absence of a correlation between trap captures and local plant aphid density suggests that winged aphids originate from sources beyond the immediate neighbouring plants, likely dispersing from greater distances across the field. This finding challenges the current understanding that winged banana aphids move primarily between proximate plants (14). Considering the relatively small trap surface area and haphazard nature of aphid landing, the observed capture rates likely underestimate true field-level flight activity. Consequently, this observed mobility pattern carries important implications for BBTD management. Critically, localized interventions targeting only individual infected mats may be insufficient, as winged aphids can potentially disperse beyond immediately adjacent mats. Therefore, management should emphasize coordinated field-level and adjacent-plot actions to limit potential vector movement and associated virus transmission risk in BBTD infected landscapes. However, the current findings are based on smaller spatial scale, thus the effectiveness of broader, landscape-level management approaches may require further validation.

Our study trials further demonstrate a 1% soap solution to significantly reduced banana aphid populations relative to unsprayed controls, suggesting that soap sprays may offer a practical, affordable, and suitable aphid and BBTD management alternative for smallholder farmers. However, comparative field studies are needed to evaluate performance of soap sprays against established insecticide. Insecticides such as imidacloprid, acetamiprid, and abamectin are currently recommended for controlling banana aphids (14). The soap spray rapidly reduced both winged and wingless aphid populations within 30 minutes to 2 hours post-application, with aphid numbers remaining low for approximately two weeks after treatment. Such biorational pesticides may also be more compatible with beneficial natural enemies, minimizing ecological disruptions within the crop ecosystem (28).

Secondly, all BBTD management protocols involving mat disturbance, such as de-trashing, infected mat roguing, and plant debris disposal (14, 29), require refinement to minimize aphid disturbance and dispersal. Our field experiments demonstrated that any plant and mat-disturbing activities trigger immediate and significant aphid movement. In the case of de-trashing, the disturbance creates a complex trade-off in BBTD management. While the removal of old leaves and sections of old leaf sheaths reduces suitable aphid habitat and prevents large colony build-up, the initial disturbance exposes concealed winged aphids, likely triggering short-term dispersal. Following exposure, winged aphids have been reported to employ defensive mechanisms, such as dropping to the ground, flight or seeking concealed areas, to evade threats (30). Similarly, infected mat removal may significantly disrupt aphid behaviour triggering dispersal. Thus field operations such as de-trashing in BBTD infected fields and removal of infected mats need to be combined with direct aphid suppression using measures such as soap spray before and/or after de-trashing to suppress winged aphid flight. Moreover, our short-term observations identified distinct daily patterns in aphid movement and activity on both undisturbed and disturbed plants. Overall aphid activity was lowest in the afternoon and evening on undisturbed plants, with reduced activity during evening hours further observed on disturbed plants. Thus, scheduling de-trashing with subsequent soap spray application, or applying soap spray prior to the removal and disposal of infected mats, during evening hours, represents an optimal integrated approach. Other farm management practices that may disturb banana plants thus affecting aphid behavior (15), such as de-suckering, sourcing suckers for planting, manuring and weeding may be advisable in the evening hours to minimize aphid disturbance and dispersal in BBTD-affected farms/landscapes.

Thirdly, the handling of infected plant debris disposal should also be optimized to minimize aphid dispersal. Aphids were observed across the whole pseudostem, including the 10 cm section just above soil level. In contrast, banana aphids were not observed on corms. Winged aphids persisted on pseudostem sections, creating ongoing transmission risk if plant debris is not properly disposed of. Cutting fresh pseudostems at soil level, just above the corm, followed by chopping the leaves and stems, and immediately burying these plant parts in pre-dug holes under a thin layer of soil, effectively prevents aphid dispersal (14, 29). Interestingly, the laboratory experiments revealed a preference for fresh leaf tissues, although field aphid colonies were mainly associated with open pseudostems and pseudostems concealed under leaf-sheath spaces. This apparent difference between field and laboratory observations may be explained by differences in ecological conditions, cryptic aphid behaviour and methodological limitations. Aphids on banana pseudostem leaf sheaths in the field are more shielded from direct rainfall, sunlight, natural enemies, and other environmental stressors compared to those on the exposed banana leaf parts while the laboratory offered uniform conditions for both tissue types. Methodologically, it was hard to maintain the hydrated state of tissues as in the field. The cut thin leaf sheaths from the pseudostems were observed to rapidly desiccate compared to the leaf tissues that remained hydrated for longer time durations. This behavioural shift highlights that both pseudostems and leaves of BBTD-infected plants require careful management and burial for optimal disposal outcomes. Once the aboveground BBTD-infected plant material is adequately disposed of (i.e. chopped up and buried), the remaining corms should be uprooted as the entire mat including the corms is systematically infected with BBTV (31, 32). The corms or corm pieces should be sliced into smaller sections, spread out on the soil in a sunny location for complete tissue desiccation. Corms or corm pieces should not be buried, as this will give rise to BBTD-infected resprouts.

Several assessments in this study used purposive selection of aphid-infested plants because the objective was to characterize aphid colony distribution, movement, survival, and response to management interventions. Although appropriate for studying aphid ecology and behaviour on colonized plants, this approach limits inference regrading overall prevalence and abundance of banana aphids across randomly selected plants and fields. A further limitation was the relatively small number of independent fields or field blocks included in several study components, generally two to five depending on the assessment. The numerous plants, traps, plant-material sections, jars, and repeated observations increased the precision of estimates under the conditions examined but did not increase the number of independently sampled field environments. In addition, the use of banana monoculture fields limits direct application/extrapolation of findings to intercropped fields or systems. Therefore, these limitations must be taken into consideration while interpreting and applying the findings of this study. Based on the findings of our study, we recommend the following refined integrated management approach of the banana bunchy top disease:

1. Regularly monitor aphid population buildup in BBTD-infected farms and landscapes to timely contain aphid colony buildup and winged aphid dispersal, especially from visibly symptomatic plants and mats. Monitoring aphid populations should involve opening up the hidden locations to check for the presence of the aphids.
2. Prompt removal and burial of pseudostems and leaves of diseased mats. BBTV is highly systemic, and all physically interconnected stems in a mat are quickly infected (31, 32). Given the high preference and survival of aphids on the pseudostems and leaves, even in harvested parts, all pseudostems in BBTD-affected mats should be cut at soil level, chopped up into smaller pieces, and buried in shallow pits covered with thin layer of soil to minimize the escape of viruliferous aphids. A thin layer of soil to cover the disposal pits should be used to minimize farmers’ labor burden. Disposal pits could be dug prior to diseased mat removal and chopping up of aboveground plant materials, so that all operations can be conducted in a matter of minutes to minimise dispersal of viliferous winged aphids. The spraying of infected mats, especially the pseudostems, with a soapy solution could be carried out before the mat removal/disposal activity. This additional step will further limit aphid movements and flight during the whole mat disposal operation. The buried and decomposed plant materials can subsequently be used as organic manure within the field.
3. Aphids were not present on underground corms. In addition, cut corms were not attractive to aphids. Uprooted corms thus offer a low risk to BBTV dispersal. Corms however need to be uprooted, sliced into smaller pieces and spread out on the soil in a sunny location to dry as buried corms will easily resprout, potentially giving rise to multiple infected plantlets.
4. De-trash potentially latently infected mats adjacent to the rogued infected mats to expose hidden aphids to environmental stressors, natural enemies and allow for easy targeting with sprays e.g. soap sprays to reduce aphid populations. Targeted spraying with soapy water on the pseudostems and into the concealed spaces, after rigorous de-trashing, ensures contact with both winged and wingless aphids, achieving better coverage and higher aphid mortality.
5. De-trashing could be applied at regular intervals, at least monthly, across whole fields, irrespective of the presence or absence of BBTD to reduce aphid population build-up.
6. When possible, schedule management practices including de-trashing and infected mat disposal operations, de-suckering, and weeding during evening hours when aphid movement is reduced.

## Data Availability

The raw data supporting the conclusions of this article will be made available by the authors, without undue reservation.
