# Supplementary material for: Banana aphid, Pentalonia nigronervosa (Hemiptera: Aphididae): ecology and implications for the integrated management of banana bunchy top disease
Source: Front Insect Sci. 2026 Aug 5;6:1883891. doi: 10.3389/finsc.2026.1883891 (PMC13488419; doi:10.3389/finsc.2026.1883891)
Supplement: Supplementary file 1 [file SupplementaryFile1.docx]

**Supplementary Information**

**Tables**

**Table S1:** Best model fits for the prediction of trapped winged aphids on sticky traps. Negative binomial generalized linear mixed model (GLMM) fit by maximum likelihood. No predictor variables reached statistical significance at α = 0.05; however, the intercept was significant (p = 0.011). Additional model diagnostics available in Figure S5.

| **Count winged aphids trapped‍** |  |  |  |
| --- | --- | --- | --- |
| Predictors‍ | Incidence Rate Ratios | CI | p |
| (Intercept)‍ | 0.21 | 0.11 – 0.41 | **<0.001** |
| Day since initiation‍ | 1.03 | 0.95 – 1.11 | 0.518 |
| Time day [2:00 pm]‍ | 1.18 | 0.79 – 1.78 | 0.414 |
| Time day [6:00 pm]‍ | 0.93 | 0.61 – 1.43 | 0.743 |
| count nonwinged aphids nearby plant‍ | 1 | 0.99 – 1.01 | 0.398 |
| count winged aphids nearby plant‍ | 1.02 | 0.88 – 1.19 | 0.768 |
| **Random Effects** | | | |
| σ^2^ | 1.51 |  |  |
| τ00 Subplot: Field | 0.51 |  |  |
| τ00 Field | 0 |  |  |
| ICC | 0.25 |  |  |
| N Subplot | 10 |  |  |
| N Field | 2 |  |  |
| Observations | 480 |  |  |
| Marginal R2 / Conditional R2 | 0.013 / 0.263 |  |  |

**Figures**

**Figure S1.**

**(1) Mapping of aphid colonies across banana plant parts and growth stages**

**Figure S2.** DHARMa diagnostic plots for wingless aphid negative binomial GLMM. Quantile-quantile plot comparing observed vs. expected uniform residuals showed no significant deviations: uniformity (KS test: p = 0.08), dispersion (p = 0.016; marginal underdispersion), zero-inflation (p = 0.07), and outlier detection (p = 0.24). The marginal underdispersion produces conservative standard errors, making hypothesis tests more stringent without invalidating the primary conclusions regarding aphid location and plant developmental stage effects on abundance.

**Figure S3**: Model diagnostics for winged aphid negative binomial GLMM using DHARMa simulated residuals. The quantile-quantile plot compares observed versus expected uniform residuals under the fitted model. Four diagnostic tests were conducted: (A) Kolmogorov-Smirnov uniformity test (p = 0.43); (B) dispersion test (p = 0.58); (C) zero-inflation test (ratioObsSim = 0.97, p = 0.62); and (D) outlier test (p = 0.28). All tests indicated non-significant deviations (p > 0.05), confirming that model assumptions were satisfied, the negative binomial distribution adequately captured the variance structure, and no zero-inflation component was warranted.

**(2) Banana aphid assessment on the lower pseudostem near the soil line to inform roguing practices**

**Figure S4**. DHARMa diagnostic Q-Q plot for aphid.counts negative binomial GLMM. Uniformity of residuals was maintained (KS test: p = 0.50). Dispersion fit was excellent (p = 0.89), and no outliers were detected (p = 1.00). The significant zero-inflation test (p < 2.2e-16; ratioObsSim = 0) reflects zero-deflation in the dataset (fewer zero counts than expected by the model) which corresponds to the sampling design ensuring aphid presence at all observed sites. This pattern does not invalidate model inference and is consistent with the biological context of the experiment.

**(3) Tracking of the field-level movement of winged aphids using sticky traps**

**Figure S5**. DHARMa diagnostic Q-Q plot for sticky trap winged aphid counts, negative binomial GLMM with nested random effects (Field/Replicate). Uniformity of residuals was maintained (KS test: p = 0.73). Dispersion fit was excellent (p = 0.85), zero-inflation test was non-significant (p = 0.97), and no outliers were detected (p = 0.76). Model assumptions were satisfied, confirming that the standard negative binomial distribution adequately captured both the variance structure and zero-frequency distribution of trap-level aphid count data.

**(4) Aphid survival on discarded banana plant material**

**Figure S6**. DHARMa diagnostic Q-Q plot for non-winged aphid counts on discarded banana plant material, negative binomial GLMM with Replicate as random intercept. Uniformity of residuals was maintained (KS test: p = 0.66). Dispersion fit was excellent (p = 0.52), zero-inflation test was non-significant (p = 0.07), and no outliers were detected (p = 0.50). Model assumptions were satisfied, confirming that the negative binomial distribution adequately captured both the variance structure and zero-frequency distribution of the data.

**(5) Testing soap spray as a low-cost aphid control**

**Figure S7**. DHARMa diagnostic Q-Q plot for soap spray experiment on live winged aphids, negative binomial GLMM. Uniformity of residuals was maintained (KS test: p = 0.14). Dispersion fit was excellent (p = 0.81), zero-inflation test was non-significant (p = 0.85), and no outliers were detected (p = 1.00). Model assumptions were satisfied, confirming that the negative binomial distribution adequately captured both the variance structure and zero-frequency distribution of the data.

**Figure S8**. DHARMa diagnostic Q-Q plot for soap spray experiment on live wingless aphids, zero-inflated negative binomial GLMM with Replicate random intercept (soap spray experiment). Uniformity of residuals was maintained (KS test: p = 0.62). Dispersion fit was adequate (p = 0.06), outlier detection was non-significant (p = 1.00), and marginal zero-inflation persisted (p = 0.024; ratioObsSim = 1.58) despite the zero-inflated specification. The residual zero-inflation may reflect structural aspects of the sampling protocol rather than model misspecification, as soap spray treatment is expected to cause mortality leading to genuine excess zeros.
